# A Global Overview of Dietary Supplements: Regulation, Market Trends, Usage during the COVID-19 Pandemic, and Health Effects

**DOI:** 10.3390/nu15153320

**Published:** 2023-07-26

**Authors:** Ouarda Djaoudene, Anabela Romano, Yasmine Djedjiga Bradai, Feriel Zebiri, Amina Ouchene, Yasmine Yousfi, Meriem Amrane-Abider, Yasmine Sahraoui-Remini, Khodir Madani

**Affiliations:** 1 Centre de Recherche en Technologies Agroalimentaires, Route de Targa Ouzemmour, Campus Universitaire, Bejaia 06000, Algeria; yasminedjedjiga.bradai@crtaa.univ-bejaia.dz (Y.D.B.); feriel.zebiri@crtaa.univ-bejaia.dz (F.Z.); amina.ouchene@crtaa.univ-bejaia.dz (A.O.); didadjaou@gmail.com (Y.Y.); meriem.amrane@crtaa.univ-bejaia.dz (M.A.-A.); yasmine.sahraoui@crtaa.univ-bejaia.dz (Y.S.-R.); k.madani@mesrs.dz (K.M.); 2 MED—Mediterranean Institute for Agriculture, Environment and Development, CHANGE—Global Change and Sustainability Institute, Faculdade de Ciências e Tecnologia, Universidade do Algarve, Campus de Gambelas, 8005-139 Faro, Portugal

**Keywords:** dietary supplements, COVID-19 pandemic, health, regulation, usage, global market

## Abstract

Over the last 20 years, the use of dietary supplements (DS) has continued to grow in many countries. Due to the public health crisis brought on by the COVID-19 pandemic and amidst fears regarding COVID-19 vaccines and their low supply in many regions of the world, there has been a marked interest in the use of DS as alternative means of protecting against and treating this emerging disease, as well as boosting the immune system and minimizing the risk of inflammation. Despite a lack of evidence to suggest their efficacy, a surge in the sales of DS has been reported in many parts of the world. Questions have also been raised about the health effects associated with DS due to their increased use during the health crisis. Numerous scientific studies have demonstrated their beneficial properties as well as some adverse and even toxic effects. In addition, given the current global interest in this issue, a review is needed to establish the status of dietary supplements before and during the health crisis. The aim of this review is to summarize the current evidence on the impact of dietary supplements on the incidence of the COVID-19 pandemic, as well as their regulation and associated market trends. First, we provide an overview of DS, including a comprehensive review of the legislative and regulatory aspects of DS in the USA, China, the EU, and Algeria. Second, we describe the prevalence of the most commonly consumed DS and their efficacy as a prophylactic modality in the era of COVID-19. Additionally, we examine the structure and size of the DS market in the countries that predominantly produce and import them, its global market trend, and the impact of the COVID-19 pandemic on market growth. Finally, in this review, we also discuss the profile of DS users.

## 1. Introduction

To promote overall health and long-term well-being, food and appropriate supplementation are required. Dietary supplements (DS) are a broad category of products that contain a “dietary ingredient”, such as vitamins, minerals, herbals, botanicals, amino acids, fatty acids, and others that may be used individually or in combination. They are meant to be consumed to supplement one’s diet and fulfill basic nutritional needs, and they are categorized according to their type or function. Although people take dietary supplements for different reasons, the most significant drivers of intake include ensuring proper nutrition, reducing the risk of age-related disorders, and protecting body tissues [1,2].

The COVID-19 pandemic, declared in March 2020, marked the beginning of many global economic and health-related issues [3]. It has spread to many countries and is wreaking havoc around the world. In addition to the severity of the disease, which affects numerous organs through immunological, inflammatory, and redox mechanisms, the use of DS, such as vitamins and minerals, which may provide some protection by boosting the immune system and helping to reduce disease severity, has emerged as a potential dietary or therapeutic adjuvant treatment for COVID-19 [4]. Despite the development of several COVID-19 vaccines and the availability of many pharmacological therapies, the demand for DS during the COVID-19 outbreak increased significantly worldwide [5,6].

Over the past 20 years, there has been a significant increase in the prevalence of supplement use. In fact, the DS market is growing in terms of sales and, more importantly, in terms of products available on the market. Consumers are being presented a large number of products, brands, and formulations, distributed through a wide variety of marketing channels [1]. The value of the global dietary supplements market was estimated to be worth nearly USD 152 billion in 2021. According to the latest STASTICA report, the global market is expected to be worth USD 300 billion by 2028 [7]. The global market for DS can be currently characterized by a continuous growth in sales, confirming the belief that they are an important part of people’s diets worldwide [8]. Research in the field has also grown, with more than 69,000 articles on DS published through PubMed between 2012 and 2022. The sales of dietary supplements increased significantly in early 2020 as a result of the COVID-19 pandemic [9]. Therefore, sales increased by 50% between 2018 and 2020, with sales exceeding USD 220 billion in 2020 [10].

As the DS market has grown and become more lucrative, the importance of ensuring product quality has increased, as have the challenges associated with this task. Therefore, the call for global quality standards and enhanced focus on the regulatory challenges associated with DS are necessary, as adulterated or mislabeled products may circumvent existing regulations, leading to an increase in the incidence of adverse reactions (of which some can be fatal) caused by contaminants or adulterants in the product rather than the ingredients in the supplements themselves [11].

In contrast to pharmaceuticals, DS are loosely regulated because they are culturally embedded and driven by an industry that is in a vacuum and separate from public health imperatives [12]. Furthermore, there is currently little agreement between countries on the scope, regulatory requirements, definition, or even the terminology that can be used to classify DS [13]. Therefore, in the interests of consumer protection and information, many countries have established regulations governing the manufacturing, importation, and sale of DS. Thus, from design to marketing, manufacturers, importers, and sellers have a clear legal framework that they must operate within, contributing towards achieving a high level of safety and consumer health protection [6].

The context within which DS are used varies widely from country to country, and they are regulated by several federal agencies and government regulations that can remove a product from the market due to contamination, misidentification, adulteration, mislabeling or false claims, post-marketing surveillance adverse event reports, and failure to adhere to good manufacturing standards [14].

Numerous barriers to the regulation of DS have serious negative implications for public health, such as inadequate safety evaluation, insufficient efficacy requirements, the poor monitoring of unsubstantiated labeling and marketing claims, inadequate quality assurance and control, and gaps in the post-marketing regulatory framework. As a result, the potential for harm from the use of dietary supplements can range from monetary loss to serious adverse health effects [15].

Dietary supplements are often bought legally to fulfill one’s nutritional needs, but some are being increasingly supplied illegally, which increases the risk of adulteration and makes them even more dangerous. However, even though a prescription is not required, a doctor should recommend and supervise their use [16].

Concerns about the use of dietary supplements include the fact that they are “unproven” and that there is insufficient data to support their widespread use. There is a critical need for reliable information. Therefore, in this review, after providing an overview of the use, definition, efficacy, and safety of DS, we will present the regulatory developments in the countries with the highest number of scientific publications on DS in order to understand the challenges they pose to the sector. For this review, we also researched the structure of the DS market in the foremost producing and importing countries. Finally, we determined the profile of DS users. The prevalence of the most commonly used DS and their efficacy as a prophylactic modality in the COVID-19 era are also discussed.

## 2. Overview of Dietary Supplements (DS)

### 2.1. Quantitative Research Literature Analysis

**Figure 1:**
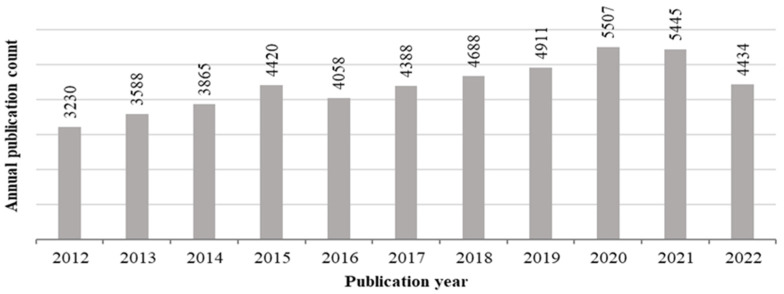
Number of publications in the last 10 years containing the keywords “dietary supplements” (generated using the Scopus online databases).

We searched the literature using the Scopus database and yielded 48,598 publications for analysis; we searched for papers on DS published between 2012 and the present day, selecting publications containing the word “dietary supplements” in the title, abstract, or keywords. In the COVID-19 pandemic period (2019–2021), the literature on this topic accumulated more rapidly (Figure 1). The top five contributors in terms of countries/territories, journals, and Scopus categories are listed in Table 1. The most productive countries were the USA and China, with 26.3% and 13.1% of the DS publications, respectively. The journal *Nutrients* was the most productive journal, and many publications were published in journals dealing with medicine and nursing, agricultural and biological sciences, biochemistry, genetics, molecular biology, and chemistry.

### 2.2. Characteristics of DS

Dietary supplements are products containing concentrated sources of nutrients or other substances such as vitamins, minerals, botanicals, algae, fungi, bacteria, synthetic products, products of animal origin, amino acids, metabolites, etc., that are intended to supplement one’s diet and have a nutritional or physiological effect, either alone or in combination with other substances. They are usually sold in dosage form, including capsules, pastilles, softgels, gelcaps, tablets, pills, sachets of powder, dropper bottles, or any other form in which these products can be ingested and taken in measured doses. In addition, their presentation, labeling, or advertising must not claim or imply that they have the ability to prevent, treat, or cure human disease, nor must the product be promoted for use as a conventional food or as the sole component of a meal or diet [14,17,18].

The lack of a global consensus on DS may be due to differences between countries in the regulatory definition and categorization of DS products. The permitted ingredients and the types of products covered by the term DS vary widely and are referred to by different names in different countries (Table 2). Differences in terminology can result in a product being classified in a completely different way, leading to discrepancies in regulatory decisions that vary significantly from country to country [13].

According to the Council for Responsible Nutrition, which tracks the sales of DS, the specific supplements that were most consumed in 2021 were vitamin or mineral supplements (98%), specialty supplements (46%; including omega-3 fatty acids, probiotics, etc.), botanicals and herbs (44%), sports supplements (30%), and weight management supplements (19%) [19].

**Table 2:** Terminology and definition of dietary supplements in different countries [13,20].

Country	Category Name	Definition
*China*	Health food (HF)	HF refers to foods that claim to have specific health functions or provide vitamins and minerals. It is specific to certain groups and modifies organic functions in humans, but is not intended to treat disease and does not cause acute, sub-acute, or chronic harm to the human body.
*USA*	Dietary supplements (DS)	DS are dietary supplements that contain one ingredient or multiple ingredients, such as vitamins, minerals, herbs or other botanicals, amino acids, and enzymes, to supplement one’s total dietary intake. They are sold in forms such as tablets, capsules, softgels, gel capsules, powders, and liquids. Unlike medicines, dietary supplements are not intended to treat, diagnose, prevent, or cure disease.
*EU*	Food supplements (FS)	FS are concentrated sources of nutrients or other substances containing a wide range of ingredients, including vitamins, minerals, amino acids, essential fatty acids, fiber, and various plant and herbal extracts, that have a nutritional or physiological effect and are available in specific dosage forms (pills, tablets, capsules, liquids) to supplement one’s normal diet.
*Canada*	Natural health products (NHP)	NHP is a category of naturally derived products such as vitamins, minerals, amino acids, probiotics, herbal and homeopathic medicines, and traditional medicines intended to improve human health (diagnosis; treatment; alleviation or prevention of a disease, disorder, or abnormal physical condition or its symptoms; restoration; modification or correction of organic functions).
*Australia*	Complementary medicine (CM)	CMs are therapeutic products consisting of one or more designated active ingredients, each of which has an established identity and a traditional use that is not of the conventional healthcare practices of a country.

### 2.3. Consumer Interest in DS and Uptake

**Figure 2:**
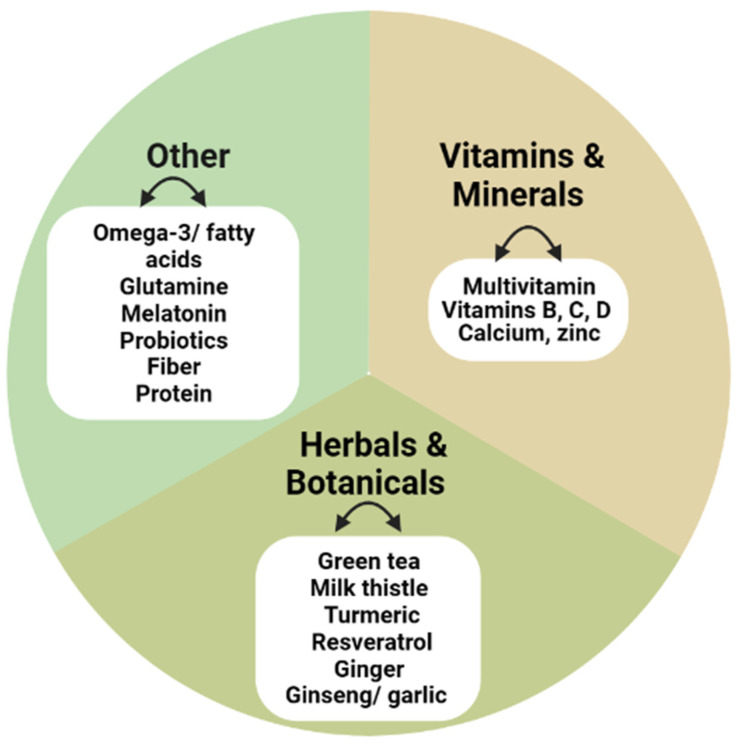
Examples of common dietary supplement categories.

Dietary supplements appear to be attractive to consumers who wish to maintain or restore a normal state of health and correct or prevent imbalances. According to the Council for Responsible Nutrition, the most-cited reasons for taking supplements are to improve immune health (36%) or “maintain” general health/well-being (44%) [20]. Despite a lack of evidence for their therapeutic efficacy, more than one third of adults in the US or Europe take a daily multivitamin/mineral supplement to prevent the development of chronic diseases. Although there is currently a plethora of supplements on the market, some of the most commonly used ones are summarized in Figure 2 [1].

DS provide concentrated amounts of nutrients with minimal calorie counts (or no calories) relative to one’s total energy requirements. Unlike foods, they can be used to improve dietary adequacy without adding significant amounts of food energy, making them useful for improving energy intake in energy-inadequate populations or high-risk groups. DS are also useful for managing specific age/life stage events such as pregnancy and for various medical conditions. Nutritional supplements are useful in helping pregnant women meet their nutritional needs, as it is extremely difficult for them to do so through diet alone. Babies need more iron, vitamin K, vitamin D, and possibly choline. Vitamin B12 supplementation is recommended, as the elderly are particularly at risk of achlorhydria. To prevent nutritional deficiencies due to malabsorption, people who have had intestinal bypass surgery need to take a variety of micronutrients such as iron; calcium; vitamins A, B, and 12; and often vitamins D and C daily [21]. In addition, DS have been widely used to prevent and treat malnutrition in populations at high risk of developing the condition; in developed countries, micronutrient powders have been used to treat deficiencies in children and pregnant women [12].

### 2.4. Safety Issues, Efficacy, and Quality of DS

The increasing use of DS raises public health concerns about their efficacy and safety in the short and long term. Issues of safety and efficacy are less common in countries where dietary supplements are regulated more like the way drugs are than in countries where they are regulated more like foods, as pre-market approval is usually required [18]. For this reason, a safety management strategy needs to be implemented before taking dietary supplements. However, it may not be wise to use DS without a doctor’s prescription [14].

The most serious safety issue posed by DS is the sale or marketing of adulterated products containing illegal and unsafe ingredients whose efficacy has not been demonstrated. This includes the failure to ensure the absence of toxic contaminants and/or pesticides, heavy metals, and active drugs in the ingredients and finished products that are not declared on the label and/or exceed the maximum doses or upper safe intakes of nutrients. Therefore, the three most problematic DS categories in the USA are sexual enhancement supplements, weight loss supplements, and sports performance/bodybuilding supplements [11,14].

Concerns about the misidentification of ingredients in dietary supplements and quality assurance/control issues remain critical for the industry and the public. In addition, the ingredients used in dietary supplements should be characterized and identified through the application of appropriate analytical methods and the development of reference standards. Several research methodologies are often required to demonstrate efficacy, ranging from basic in vitro studies on mechanisms of action to animal and human studies. In addition, there is an urgent need for more (and better) clinical research on the efficacy and safety of DS with respect to health outcomes [11].

The issue of safety, efficacy, and quality is somewhat challenging because there is a wide range of variations in DS in terms of source, physicochemical properties, and dosage form, especially when these products are combined [1]. However, databases of DS for public use are needed to develop uniform, common definitions to identify content, evaluate interventions, and assess the contribution of DS product formulations to health. This can be achieved by investing in and exploiting advances in databases, software and data science technology [21].

## *3.* Legislative and Regulatory Issues

DS manufacturers follow a number of guidelines to ensure the production of products with real value. Numerous regulatory bodies around the world develop strict, comprehensive rules and standards to ensure consumer effectiveness and safety. Regulatory organizations may also consider whether the products provide value to the consumer. Due to restrictions that vary from country to country, exporting DS is challenging, especially if the regulations are unclear or the products have not been updated to comply with the regulations of the importing country (Table 3).

### 3.1. Regulation in the United States of America (USA)

The Federal Food and Medications Act of 1906 was the first from of federal regulation regarding food and drugs in the USA, providing definitions of “adulteration” and “misbranding” and giving the federal government the power to penalize those who produced such products [24]. The first DS appeared in the USA in 1920, consisting mainly of nutrients and food ingredients [25]. Years later, laws regulated their production, composition, labeling, and distribution. Today, DS are regulated by the Food and Drug Administration (FDA) through the Dietary Supplement Health and Education Act (DSHEA) of 1994 [26]. Both DS products and their ingredients are regulated as foods. A dietary supplement is not intended to treat, prevent, or cure any disease. The FDA’s primary responsibility is to ensure the safety and purity of DS after they are marketed and to remove any product that may be potentially dangerous to the consumer from the market [25,27].

Since 1997, the FDA has maintained a list of regulations governing good manufacturing practices for DS. The final rule “21 CFR part 111” was published in 2007 and includes good manufacturing and distribution practices, labeling, packaging, and record-keeping requirements. Tracking the product to verify its integrity, quality, and safety from manufacture to distribution is a key component of good manufacturing practices. Five statements must be made on the label: The first pertains to the identification of the product; a dietary supplement must be labeled as a “dietary supplement” or provide an indication of the ingredient(s) intended to supplement one’s diet. Secondly, nutritional information; the name and address of the manufacturer, packer, or distributor; a declaration of the ingredients; and finally the exact contents must be disclosed on the label. Manufacturers are required by law to keep a record of all adverse event complaints and to report serious adverse events to the FDA within 15 days of receipt. A home address or telephone number must also be listed on the labels of DS products so that anyone can report an adverse event. Manufacturers are also required to keep records of all adverse event reports they receive for a period of six years and make these records available for review by the FDA [28,29]. In addition, in the USA, DS do not need premarket approval, but the manufacturer must guarantee that the products are safe [13]. To do this, information on ingredients and safety must be provided in the following ways: through the New Dietary Ingredient Notification (NDIN) process, the addition of any new dietary ingredient(s) into a DS product must be disclosed to the FDA 75 days before it becomes available on the market [29]; however, if an ingredient was used in a product before 1994, it is grandfathered and may continue to be used without the manufacturer having to notify the FDA [30].

In the USA, more than 50% of people take at least one DS; however, these products are usually intended to be taken orally only and cannot be recommended for use by any other routes of administration [13]. The FDA receives many reports of unexpected events such as hospitalization, allergic reactions, congenital abnormalities, etc. Denham [31] reported that more than 23,000 people in the USA suffer from the adverse effects of DS each year. Despite resistance from the industry, the FDA continues to make significant efforts to address the issue [28].

### 3.2. Regulation in China

In 1996, the Ministry of Health (MOH) approved health foods in China for the first. In 2003, the China Food and Drug Administration (CFDA) took over the regulation of health foods from the MOH. Since then, the CFDA (renamed after the restructuring of the Chinese cabinet in 2018) has been the Chinese regulatory authority for drugs and medical products and part of the State Administration for Market Regulation (SAMR). Its objectives include the development of standards and categorization systems for medical devices, as well as laws and regulations for drugs, cosmetics, and medical devices [32].

In 2005, the MOH’s original definition of health foods was expanded and divided into two categories: vitamin and mineral supplements and functional health foods. The CFDA published a list of the recognized health benefits of health foods, including boosting immunity, antioxidant activity, memory enhancement, reducing eye fatigue, improving sleep, facilitating digestion, etc. [33].

The 2015 Food Safety Law classifies health foods as a special category of foods that are strictly regulated and controlled by the government. Several articles in China’s Food Safety Law discuss the various requirements of health foods. The Quality Standard of Health Food Registration in China was established based on the GB 16740-2014 standard. Its main purpose is to provide the bare minimum guidelines, requirements, and necessary instructions. For example, it includes rules for the approval of claims, ingredients, the registration and submission of domestic and imported products, labeling, the sensory testing of the product, physical and chemical testing, usage requirements for the use of vitamins and minerals, and requirements for the quality of ingredients [12].

### 3.3. Regulation in European Union (EU)

In Europe DS are regulated by the European Commission through several directives. The European Commission has established continent-wide regulations to protect consumers and provide them with safe food [34]. However, the regulatory framework for DS in Europe follows general food legislation and their manufacturing processes are subjected to Good Manufacturing Practices (GMP) and Hazard Analysis Critical Control Point (HACCP) procedures [35,36]. To ensure the integrity and safety of dietary supplements and the safety of consumers, the scientific Committee on Food (SCF), currently the European Food Safety Authority (EFSA), has established upper tolerable intakes (UTIs) of minerals and vitamins that can be ingested daily to support and maintain good health [34]. A variety of nutritional components other than vitamins and minerals may be used in the manufacturing of food supplements once they have been approved by the regulatory body (EFSA). Under Directives 2002/46/EC and 2001/15/EC, these substances can be classified as amino acids, enzymes, prebiotics and probiotics, essential fatty acids, and botanical extracts. In addition, the aforementioned directives established uniform guidelines for the labeling of food supplements, and these guidelines outline the requirements for specifying the identity and composition of DS products [36].

While the nutritional value of certain ingredients may be outlined on a product’s label, the amount of vitamins and minerals, followed by the maximum recommended daily intake, storage conditions, and the risk associated with excessive consumption of the product must be stated. Therefore, DS must not make therapeutic claims or refer to conventional diets. These features provide consumers with general information and protect them from fraudulent advertising [37].

The legislative texts under Directive 1925/2006/EC allow for the prohibition or exclusion of certain ingredients, other than vitamins and minerals, to be added to foods, including food supplements that may have undesirable effects on the consumers. A positive list of safe substances should be established to ensure the proper functioning of the internal food supplements market.

Meanwhile, under Directive 2015/2283/EC, specific legislative measures apply to novel foods that have not been used in the European Community before 15 May 1997. This directive has led to the establishment of an appropriate list of the novel ingredients that may be used in the production of DS to ensure their access to the EU market [13,37].

Regarding the internal market, the Directorate General for Health and Consumer Protection (DG-SANCO), accompanied by the competent authorities of the member states and the EFSA agency, monitor and control the European dietary supplement market based on the Rapid Alert System for Food and Feed (RASFF) databases, which require important information related to food safety, including functional foods, fortified foods, and dietary supplements. Therefore, it is important to note that DS do not require pre-market authorization or prior safety assessments [38,39]. In fact, the European Commission has established the concept of mutual recognition to ensure the free movement of DS between member states. It offers all products legally manufactured in one member state the chance to be sold without restrictions in another member state, even if these products are supposed to be subjected to different national regulations. Currently, some member states require a note of authorization to place a DS on the market [13].

### 3.4. Regulation in Algeria

To date, there is no strict regulatory framework in Algeria that applies exclusively and specifically to DS. They are subject to the regulations governing the commercial activity of foodstuffs (consumer control and protection) and do not require authorization from the Ministry of Trade and the Ministry of Health for their production, importation, or marketing. DS are over-the-counter products that can be acquired without a medical prescription and, unlike drugs, do not require authorization prior to being made available on the market [40,41].

The Ministry of Trade takes action in the event of a warning from the appropriate authorities regarding product withdrawals or anomalies in the dietary supplement market. The risks of dietary supplement consumption are related to the presence of toxic, banned, and unauthorized substances in the supplement’s ingredients. There have been numerous cases of fraudulent advertising with respect to DS in Algeria, and many dietary supplements have been withdrawn from the market [42].

In addition, in 2022, the Ministry of Trade and Export Promotion announced a ban on 20 dietary supplements that had been tested in laboratories and found to contain chemical components used in the pharmaceutical industry rather than those declared in the products’ composition; thus, these products were deemed potentially harmful to consumers’ health. These supplements mainly contained drugs used to treat sexual impotence, and consuming them without consulting a specialist is not recommended [43].

Since the health crisis, the market for DS has grown, meaning that a regulatory framework is urgently needed. These risks require increased regulation. Therefore, DS need a specific regulatory framework that gives them a real status to ensure consumer safety and combat false advertising and poor manufacturing practices [44].

## 4. Trends in the Use of DS before and during the Emergence of the COVID-19 Pandemic

Consumers have become much more concerned about their health in recent years due to the rapid development of societal medical knowledge and living standards. Additionally, the coronavirus pandemic’s emergence has increased everyone’s awareness of the importance of health and the need to maintain one’s health. This infectious disease has had a significant impact on lifestyles and the global economy. Therefore, finding ways to combat or mitigate the effects of this disease is imperative. Dietary supplements with specific health benefits and/or the potential to regulate bodily systems have steadily attracted the attention of consumers. A healthy diet and DS have gained attention as potential co-adjuvants in managing and preventing COVID-19. Hence, there are important public health reasons for taking some supplements, including vitamins C, D, and B, zinc, and probiotics, in order to boost one’s immunity [45,46].

The aim of this section was to assess changes in the consumption of DS, with a particular focus on the supplements affecting immunity during the worldwide COVID-19 pandemic.

The FDA treats DS as foods and considers them to be drugs. Therefore, unlike prescription drugs, they do not have to be proven safe or effective before they are made commercially available. Although they are intended to be consumed in various forms (capsule, tablet, powder, liquid, etc.), they are derived from natural sources and meant to boost immunity against diseases such as viral infections, inflammation, and respiratory complications. Most commonly, they are used as adjuvant treatments or additives in conjunction with a main pharmacological drug [4,47].

### 4.1. Characteristics of the Consumption of DS

Sales of DS and nutraceuticals increased during the pandemic due to their perceived “immune-boosting” effects. However, little is known about the efficacy of these DS against the novel coronavirus or the disease that it causes, COVID-19 [9]. Here, based on the evidence available in the literature, we provide a comprehensive assessment of the potential preventive and therapeutic value of different DS. These include zinc and vitamin C and D, which are often used by the general public to prevent or cure respiratory infections or support immune health.

The use of DS is widespread in the USA, China, and several European countries. Several studies have investigated the association between the use of different types of DS and the risk of COVID-19; seemingly, there was a trend wherein the use of DS during the pandemic increased [10,48,49,50,51]. According to these cross-sectional studies, the prevalence of supplement use is associated with several factors, such as socio-demographic background, health and lifestyle characteristics, changes in the reasons for dietary supplement use, and changes in the type and circumstances of dietary supplement use.

The increased use of DS during the pandemic has been reported in several studies (Table 4). During the COVID-19 pandemic, an increase in the rate of supplementation was observed, with a 40% increase in the consumption of vitamin C [51], 82% increase in the consumption of multivitamins [50], and 23% increase in global intake [49]. Differences in the prevalence of use of these products are related to the socioeconomic level of each country, the level of knowledge of the benefits and harms of these supplements among different populations, and the influence of the media [10,49].

### 4.2. Consumption of Supplements According to Pandemic Status

COVID-19 affects the immune system, causing a systemic inflammatory response or cytokine release syndrome. High levels of pro-inflammatory cytokines and chemokines have been found in COVID-19 patients [53]. COVID-19 is also associated with the development of microthrombi and coagulopathy, which can later lead to sepsis, acute respiratory distress syndrome, and secondary infections [9,54].

During the COVID-19 pandemic, several vaccines and drugs were tested for efficacy, safety, and dosage, which took a long time to validate. Researchers are also looking for alternative strategies to prevent the disease. In addition to herbal remedies and nutraceuticals, DS offer patients a promising preventive treatment option. This approach could help strengthen the immune system and further suppress hyper-inflammation, providing both prophylactic and therapeutic support against COVID-19 [4,55].

Many research efforts have been devoted to explaining the role of nutritional supplementation in the prevention and management of COVID-19 infection. The use of DS, such as vitamin and mineral supplements, has emerged as a putative nutritional or adjunct treatment approach for COVID-19. Vitamin and mineral supplementation is critical during COVID-19 in order to reduce the severity of symptoms and shorten the duration of respiratory infection, thereby improving immune responses. There are research reports showing an increased interest in DS such as vitamins (C, A, E, and D), zinc, omega-3, probiotics, selenium, and others during the COVID-19 pandemic (Table 5) [10,46]. In addition, several authors have highlighted that vitamin C and D and zinc were the dietary supplements that were most commonly used to boost immunity and reduce the risk of acquiring COVID-19 [6,56].

Given the severe lack of specific therapies for COVID-19 infection, medical recommendations have promoted the idea that certain vitamins, particularly vitamin C and D, are the most effective elements for the prevention and treatment of COVID-19. There are numerous reviews and studies discussing the role of vitamins (especially C and D) against COVID-19 transmission (Table 6). Indeed, vitamin D is known to have immunomodulatory, anti-inflammatory, antioxidant, and antiviral properties [55,57].

Vitamin D, a secosteroid hormone, was thought to be able to reduce the risk of COVID-19 during its critical period by stabilizing physical barriers, regulating the renin-angiotensin system, and enhancing cellular innate and adaptive immunity [58]. Appropriate supplementation may boost one’s immune system. Cangiano et al. [59] examined the mortality rate following the spread of COVID-19 in 157 residents of a nursing home in Italy and reported that the mortality rate was inversely proportional to vitamin D supplementation. According to a cohort study by D’Avolio et al. [60], significantly lower 25-hydroxyvitamin D (25(OH)D) levels were found in PCR-positive SARS-CoV-2 patients compared with negative patients. Furthermore, the role of vitamin D in reducing the risk of COVID-19 has been confirmed in several observational studies and clinical trials. Therefore, supplementation with vitamin D could be recommended to vitamin D-deficient COVID-19 patients [61,62,63,64].

Vitamin C has also been proposed as a possible nutritional intervention for COVID-19. Indeed, vitamin C is a potential therapeutic candidate for the prevention and treatment of COVID-19 infection as well as an adjunctive therapy in the intensive care of COVID-19 due to its long history of use against the common cold and other respiratory infections and its many beneficial properties (anti-inflammatory, immunomodulatory, antioxidant, antiviral, and antithrombotic) [4,9]. The vitamin has effector pathways in both the innate and adaptive immune systems and has direct virucidal activity. Regarding the critical phase of the COVID-19 pandemic, vitamin C helps to control and reduce cytokines, protects the endothelium from oxidative damage, and is crucial for tissue healing [65]. Several studies have reported improved clinical outcomes following vitamin C-involving treatment, mainly with respect to shorter hospital stays and a reduced need for mechanical ventilation or earlier resolution of symptoms [66].

**Table 5:** The most popular dietary supplements for COVID-19 prevention.

Most Used DS (%)	Vit. D	39.0	55.7	15.3	31.6	49.1	60.2	50.1	52.5	31.1	30	22.4
	Vit. C	19.4	77.8	11.4	84.5	26.6	31.4	30.1	27.0	-	-	23.0
	Vit. B	-	14.1	9.1	9.4	-	-	-	-	-	-	6.2
	Multivitamin	27.4	21.9	16.6	17	43.9	58.3	41	-	-	14	12.6
	Zinc	15.8	42.9	5.7	8	12.4	13.4	17.8	17.4	1.8	9	30.4
	Selenium	-	19.3	-	-	-	-	-	13.1	-	-	2.5
	Omega 3	81.9	-	8.6	11.7	22.4	26.8	22.6	25.0	-	-	25.5
	Probiotic	22.3	-	4	4.4	11.6	22.5	12.8	4.5	20.9	-	-
Country		Turkey	Middle East	Turkey	UAE	UK	USA	Sweden	Poland	Poland	Tehran	Egypt
Number of Participants		550	2100	488	2060	372,720	45,757	27,373	3274	935	510	400
Type of Study		Cross-sectional	Cross-sectional (web survey)	Cross-sectional	Cross-sectional inquiry	App-based community survey			Cross-sectional (web survey)	Survey (questionnaire)	Cross-sectional inquiry	Cross-sectisectional
References		[67]	[6]	[68]	[69]	[46]			[10]	[70]	[71]	[72]

Some authors even recommend oral supplementation with 1–2 g/day of vitamins in order to alleviate the transition to the critical phase of COVID-19 [73]. A clinical research report in the US found low serum levels of vitamin C and D in most critically ill COVID-19 ICU patients. Older age and low vitamin C levels appeared to be co-dependent risk factors for mortality [74].

Of all of the mineral supplements studied, zinc has emerged as a leading prophylactic and therapeutic candidate against SARS-CoV-2. Zinc is a key trace mineral that is essential for both innate and acquired immune responses to viral infection. It is also involved in many biological processes, including immunity [53]. In fact, zinc contributes to the activation of the antiviral immune response by stimulating the synthesis of pro-inflammatory cytokines, including interferon and acute phase reactants, as well as promoting the proliferation of cells involved in the innate and adaptive immune systems [75]. Therefore, these DS can be used as complementary forces in the treatment of COVID-19 through various mechanisms, providing substantial support with respect to individual immunity, inhibiting SARS-CoV-2 RNA replication, and preventing virus entry into cells. Therefore, Zn may also be critical in reducing the exaggerated inflammatory response, risk of pneumonia, and duration of illness [76,77].

An observational study found that zinc levels were significantly lower in COVID-19 patients than in healthy controls and that these patients were more likely to have complications and longer hospital stays than non-zinc-deficient COVID-19 patients [78]. In a related study, samples taken from COVID-19 patients who died were shown to have lower plasma zinc levels than those taken from patients who survived the virus [79]. A high dose of oral zinc salt resulted in clinical recovery, improved oxygenation, and reduced shortness of breath among COVID-19 patients [80].

There is sufficient evidence to suggest that DS could be a good strategy to help reduce the adverse effects of COVID-19. Nevertheless, some dietary supplements may be subject to limited regulation by authorities, and consumers should be wary of misleading information and false promises surrounding them. However, it is important to note that medical advice should be sought before taking DS to reduce potential adverse effects. Excessive vitamin and mineral supplementation can cause adverse and even toxic effects and gastrointestinal tract disorders. Otherwise, interactions between supplements and drugs should be considered in terms of increasing toxicity and drug efficacy.

**Table 6:** Health benefits and potential mechanisms of the DS widely used to fight against SARS-CoV-2 infection.

Nutrient	Health Benefits	Mode of Action against SARS-CoV-2	References
Vit. D	Support immune system; immunomodulating, anti inflammatory, and anti-infectious role	– Reduce cytokine storm syndrome; – Induce the production of cathelicidin and defensins, which reduces the survival and replication of the virus; – Increase the level of soluble angiotensin-converting enzyme 2 (ACE2), inhibiting the virus from entering the cells; – Increase the level of soluble angiotensin-converting enzyme 2 (ACE2), inhibiting the virus from entering the cells; – Prevent the accumulation of angiotensin II and decrease its pro-inflammatory activity; – Reduce the risk of injury to various tissues/organs, as well as the mortality and severity.	[81,82,83,84,85]
Vit. C	Antioxidant, anti-inflammatory, antiviral, immunomodulatory, and anti-thrombotic effects; pleiotropic function	– Epigenetic regulation of various genes (up-regulation of antioxidant proteins, down-regulation of pro-inflammatory cytokines rather than direct oxidants scavenging); – Counteracting the actions of pro-inflammatory cytokines, especially IL-6; – Decrease inflammatory markers such as ferritin and D-dimer and attenuate the excessive activation of immune responses; – Inhibit endothelial surface selectin expression and platelet–endothelial cell adhesion; – Reduce D-dimer levels, which is an important indicator of thrombus formation; – Prevent the hyperactivation of immune cells; – Suppress cytokine storms, improve pulmonary function, and reduce the risk of acute respiratory distress syndrome.	[4,9,65,86]
Zinc	Immunomodulatory and antiviral properties	– Inhibit the synthesis, replication, and transcription complex of coronaviruses; – Suppress cytokine storm; inhibit SRAS-CoV-2 entry into the host cell; – Reduce organ damage secondary to the inflammatory response to SARS-CoV-2.	[53,75,76,77,79,87]

## 5. The Market for Dietary Supplements in Producer and Consumer Countries and the Impact of the COVD-19 Pandemic

### 5.1. Distribution Channel

Dietary supplements are distributed through a variety of channels, including offline distribution channels such as pharmacies, drugstores, supermarkets, specialty stores, independent retailers, and other direct-to-consumer channels. Due to their greater popularity, supermarkets and hypermarkets accounted for approximately 33.9% of all dietary supplement sales in the offline sector in 2021. The offline sector dominated the market, accounting for 81.0% of total sales, as it is easily affordable [88]. However, the internet, mobile communications, and social media have all given rise to new technology-based communication channels, known as “digital channels” or “electronic channels”, which consumers generally use to gather information about DS [10]. In addition, the proliferation of COVID-19 has attracted more customers to extensive e-commerce platforms, making many supplement brands easily accessible to consumers.

### 5.2. DS Global Market

**Figure 3:**
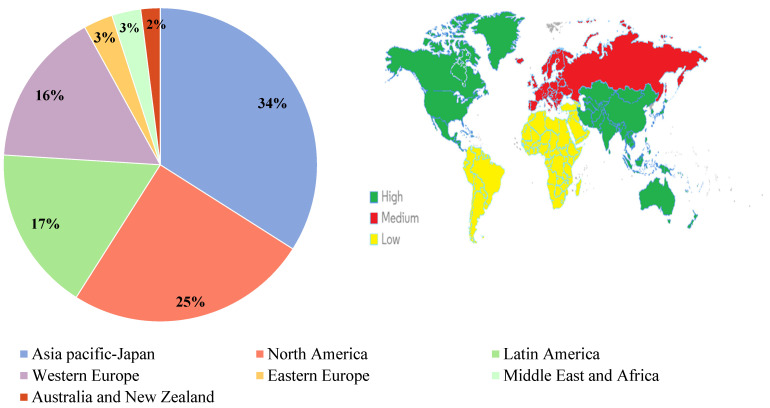
Geographical distribution of dietary supplements market and level of DS market growth worldwide (High, Medium, and Low).

**Figure 4:**
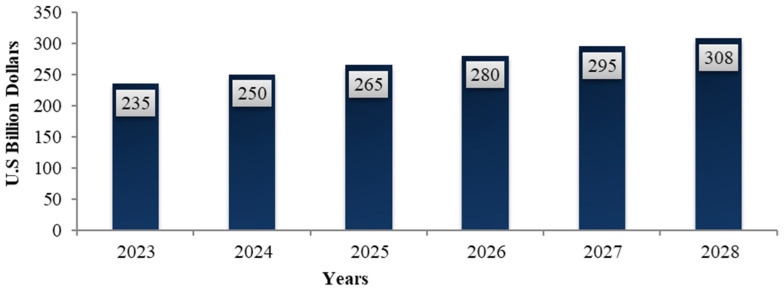
DS market size growth forecast for the period 2023–2028 [94].

The global DS market is growing exponentially in most countries. According to data from the National Health and Nutrition Examination Survey (NHNES), it has been gaining interest since the 1970s [1,89]. The geographical distribution is highly variable and has been divided into six major regions (Figure 3) [35,90]. The USA, Europe, and Japan account for the largest share of the market, followed by Asia, Australia, and Oceania, all of which demonstrate the expansion of the market [8]. In contrast, the Middle East and Africa have seen a surge in DS sales, with South Africa remaining the most important market [91]. In the Middle East, the demographics and socioeconomic status of consumers, particularly in Saudi Arabia, has led to the development of this market, which is estimated to reach SAR 875 million by 2021 [92]. Differences in these geographical distributions may be the result of several factors, such as socio-demographic and socio-cultural characteristics, consumer behavior, and ageing populations. Globally, the market size of DS increased from USD 82 billion to USD 149.50 billion in 2021 [10,37,88,93], and this will supposedly rise to approximately USD 181.2 billion in 2022 and reach USD 308 billion in 2028, with a compound annual growth rate growth of 8.90% (Figure 4) [94].

### 5.3. Market Size of DS in USA and EU

The demand for nutritional products increased significantly in the US during the COVID-19 pandemic. In 2019, global sales reached USD 345 million, an increase of 5% from 2018, and multivitamins remained the best-selling category, with nearly 120 million units sold [93]. The Council for Responsible Nutrition (CRN) survey reported increases of 59, 44, and 37% in the use of multivitamins, vitamin C, and vitamin D, respectively, among US residents aged between 18 and 35 (47% men vs. 39% women).

Regarding the European market, it has experienced strong growth since 2020 compared to previous years (EUR 7.1 billion in 2015); the annual share was estimated to be USD 14.95 billion in 2019 and is expected to increase by 9.3% CAGR to reach an expected USD 33.80 billion in 2027 [88,95]. DS sales exceeded EUR 1600 million in Italy and EUR 1 billion in Russia, while the market was worth more than EUR 100 million in other countries (including Germany, the United Kingdom, France, Poland, Norway, Finland, Sweden, Belgium, Spain, the Netherlands, and Hungary). On the other hand, in countries such as Macedonia, Georgia, Estonia, and Denmark, sales values ranged from EUR 4.4 million to EUR 98.7 million. Thus, vitamins and minerals have the highest turnover, followed by proteins, enzymes, fatty acids, and others [8,95].

### 5.4. Market Size of DS in the Middle East and Africa

In regions such as the Middle East and Africa, the DS market is constantly changing, and it especially changed during the COVID-19 pandemic. In the United Arab Emirates, for example, the market had grown by 7% by 2019 [96]. However, the demand for DS is expected to continue to grow among the Arab population, with a multinational study showing that the use of dietary products containing vitamin C, vitamin D, and zinc is even higher in Middle Eastern Arab countries (at 77.8%, 55.7% and 42.9%, respectively). About 80% of the Jordanian population continues to use DS and herbs [6,97]. Unfortunately, there is a lack of scientific articles assessing their market share and consumption in Africa.

A study by Bayazid et al. [49] showed a significant increase in the consumption of DS among the Algerian population, including omega-3 fatty acids, zinc, magnesium, selenium, cloves, ginger, turmeric, and vitamin C and D. The prevalence of DS users was 63.4%, increasing during the pandemic compared to previous years (29.4%). From a socioeconomic perspective, there are statistically significant differences between countries in the frequency of DS use. This variation is related to several factors, particularly the demographics of those in the countries [49].

### 5.5. Economic Impact of DS

DS is not only good for our health but it can also be very exciting from an economic point of view. The DS sector is a major economic driver in the countries where DS are predominantly produced, providing a significant number of high-paying jobs. The industry employs 750,000 Americans and generates USD 5.75 billion in state taxes in the US [98].

The evolution of DS prices, production rates, consumer demand for health and wellness, performance, research, and scientific production at various times, including the health crisis brought on by COVID-19, reflects the impact of DS on various sectors in general and the global economy in particular [99,100].

As soon as the COVID-19 pandemic broke out, recommendations were made to take certain DS; hence, their sales increased. In addition, the turnover generated by DS has increased from USD 4 billion in 1994 to USD 55.8 billion in 2020 [101]. According to Bayazid et al. [49], sales increased from USD 101.38 billion in 2018 to approximately USD 220.3 billion in 2020.

In all sectors, scientific research is a key driver of productivity, which is part of economic growth. Basic research informs applied research, which, in turn, drives technological advances that increase productivity. Therefore, scientific research is linked to economic growth because it influences technological progress and increases public awareness of the potential effects of DS, which, in turn, increases consumer demand [102,103]. Over 30,000 and 48,598 scientific papers on DS are listed in the Scopus and PubMed databases, respectively. The development of scientific research on health and wellness products increased significantly during the health crisis brought on by the pandemic. There are very few studies and research papers on DS in Algeria, with only 68 documents published in Scopus to date.

The economic impact of DS in Algeria is only now beginning to be seen, as the DS industry is relatively young compared to other global markets. Crucially, the growth in DS market could lead to the development of new businesses in the sector, which will help to create jobs and reduce the rate of imports, thus contributing to the economic development of countries. Recently, the Algerian market for DS health and wellness products has experienced remarkable growth [104].

## 6. Consumer Profile of DS

**Figure 5:**
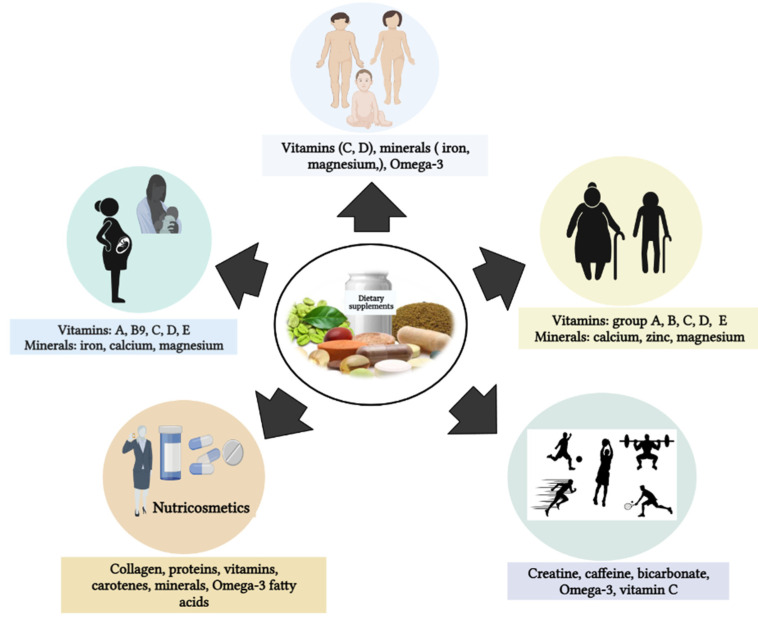
Summary of the consumer profile of dietary supplements.

The physiological or nutritional role of DS lies in prevention, maintaining good health, relieving everyday discomfort, and restoring the physiological balance of the human body. In addition, DS consumption has focused on wellness, health, and beauty. As a result, DS use has increased worldwide. Data from the NHANES series showed that, between 2007 and 2018, DS use increased rapidly. The reasons for this trend may be related to the increasing interest in supplementation among different categories of users (Figure 5) [18].

### 6.1. Pregnant and Lactating Women

Many women who were pregnant or breastfeeding used at least one supplement during their pregnancy. According to Jun et al. [105], about 50% of pregnant women and 40% of breastfeeding women took supplements after receiving advice from a healthcare professional.

Pregnant women should be advised to focus on consuming a balanced diet and eating important sources of certain nutrients. However, the use of supplements may reduce the risk of adverse effects and be beneficial in some circumstances. Supplements such as folic acid, iron, and vitamin B12 are essential for the growth and delivery of healthy babies. In addition, different cohorts of studies from the USA, Canada, and Australia show that the use of supplements is very common in pregnancies with a higher risk of nutritional deficiencies and a high burden of pregnancy complications as a way of reducing the risk of outcomes such as pre-eclampsia, gestational diabetes mellitus, and small-for-gestational-age babies, among others [106]. Nevins et al. [107] reported that supplementation with omega-3 fatty acids during pregnancy and lactation may benefit the cognitive development of children. A study by Tang et al. [108] showed that Chinese women who were pregnant appeared to take DS frequently, and about one third of them continued to do so even after giving birth. The three most commonly used supplements are calcium, folic acid, and iron.

### 6.2. Older Adults

The use of DS among older people has increased over the years, and this may be related to a number of factors, including perceived health-promoting properties, over-the-counter availability, and a general belief that DS are natural and therefore safe. The main reason older people use different DS is to reduce their risk of developing age-related chronic diseases such as cancer, CVD, and cognitive impairment. However, more in-depth research is needed to assess the safety and confirm the efficacy of DS use among older adults [109].

### 6.3. Children and Infants

In recent years, DS have been widely administered to infants and children. Vitamins and supplements for bone and tissue repair are increasingly being used among this user category [1]. In addition, it has been reported that 12–89% of parents use DS to treat hyperactive/attention deficit disorder, asthma, colds, cancer, infantile colic, and epilepsy in their children. Reportedly, children have shown positive outcomes following the intake of normal doses of multivitamins, omega-3 fatty acids, and other supplements [110].

However, parents should be aware of the risks of overdosing, intoxication, and adverse reactions when giving DS to their infants or children. Therefore, a survey and/or list of the possible adverse effects of dietary supplement consumption is needed.

### 6.4. Athletes

Sports supplements are widely used by adults and especially by athletes. Over the past 20 years, sports supplements have become a mainstay among athletes. Sports supplements are used for a variety of reasons, mainly to enhance health, hasten recovery, and improve performance during competitions and high-intensity training sessions. In addition, some studies have described gaining muscle mass and losing body fat, increasing energy, delaying fatigue, and restoring nutrients as motivational factors for DS use among athletes, all of which ultimately lead to improved performance [111]. The use of DS may be necessary when dietary intake or food preferences are restricted, or DS may be used as temporary remedies when a deficiency syndrome has been identified [112]. In addition, the majority of available evidence supports the beneficial effects of some supplement ingredients, such as creatine, β-alanine, and bicarbonate, on some types of activity (e.g., they appear to be effective in combat sports), and also others, such as caffeine, omega-3 fatty acids, vitamin C, D, and B12, and polyphenols [111,113,114].

The use of sports supplements has increased among athletes, but it has also spread to the general public. Therefore, the expansion of the sports supplement market is driving the development of standards, laws, and regulations that need to be standardized internationally for the sake of consumer safety [111].

### 6.5. Others

Natural products that can improve health and beauty are increasingly in demand today. Nutricosmetics are the latest trend in the cosmetics industry, driven by greater consumer awareness of aesthetic needs, skin aging, and wrinkles. However, many nutritional supplements intended to maintain the natural beauty of skin, nails, and hair, such as collagen, peptides, proteins, vitamins, carotenes, minerals, and omega-3 fatty acids, are considered effective due to their historical use [115].

## 7. Conclusions and Prospects

Dietary supplements are a health tool; they are designed to improve well-being and support physiological functions. By supplementing one’s diet, DS can fill gaps and corrects imbalances, which, in the long term, improves one’s health and protects against disease. Preventing and treating disease without drugs has become a global trend among consumers and healthcare and medical professionals. At the time of the COVID-19 health crisis, the market of DS grew rapidly and continuously. As a result, the number of users has been steadily increasing. In order to clarify the issues associated with the dietary supplement market, in this review, we have tried to answer several questions by giving an overview of DS and their regulation in different countries.

The everyday consumption of DS is possible due to their availability in supermarkets and convenience stores and their food status. In addition, their limited regulatory (labeling) and legislative requirements, compared to medicines, have also facilitated their widespread consumption.

The dietary supplement industry is huge, so DS are sold and supplied by multi-million dollar companies with large profits. The production of supplements is fairly straightforward, with minimal regard for safety, quality, and efficacy, as they are not subjected to strict regulation. Various supplements have been developed in recent years, but progress in this area has been slow. Awareness of the need to regulate the dietary supplement sector has led food and drug authorities to take steps to standardize regulations to improve consumer safety. With the exponential worldwide growth in the use of and demand for dietary supplements, there is a need for large-scale studies to establish their efficacy and safety.

The market for DS is clearly growing in Algeria, and unlike prescription drugs, these products are developing without any control or regulation. Many worry that this lack of regulation may have a negative impact on consumers, especially as some DS are considered to be drugs. Therefore, discussions are underway to establish a regulatory framework for the marketing, manufacturing, and labeling of DS, and demand for greater scrutiny regarding manufacturer’s health claims form the basis for the regulation of DS.

Dietary supplements are different from foods and pharmaceuticals, and given their widespread use, it is important to develop compositional databases to track their contribution to the intake of nutrients and other bioactive constituents and assess the effects these substances have on human health. There is also a need for greater collaboration across government agencies in their development.

In general, there is a growing societal interest in the use of DS; therefore, research on the issues and important aspects related to them must accompany the expansion of their use. In conclusion, the best advice is to use DS only in specific cases of public health crises or in the case of medical necessity. The ideal strategies for a long and healthy life are a healthy diet rich in fruits and vegetables. 

## Figures and Tables

**Table 1:** Top five contributing countries, journals, and Scopus categories of the dietary supplement publications.

Contributor	Publication Count (% of Total)	
*Country/Territory*		
United States	12,797 (26.3)	
China	6383 (13.1)	
United Kingdom	3571 (7.3)	
Italy	3152 (6.5)	
Canada	2579 (5.3)	
*Journal*		
Nutrients	2769 (5.7)	
Poultry Science	1120 (2.3)	
Journal of Animal Science	1012 (2)	
PLOS One	909 (1.9)	
Journal of Dairy Science	753 (1.5)	
*Scopus category*		
Medicine	25,160 (51.8)	
Agricultural and Biological Sciences	14,558 (29.9)	
Biochemistry, Genetics Molecular Biology	12,776 (26.3)	
Nursing	11,617 (23.9)	
Chemistry	3892 (8)	

**Table 3:** Regulations/laws and regulatory requirements of dietary supplements in different countries (USA, EU, China, and Algeria) [13,22].

		USA	EU	China	Algeria
Regulatory agency		Food and Drug Administration (FDA)	– The European Commission and the competent authorities – EFSA (European Food Safety Authority), if centralized procedures apply	– CFDA (China Food and Drug Administration)	NA
Regulation and law		– FD & C Act (Federal Food, Drug, and Cosmetic Act) – DSHEA (Dietary Supplement Health and Education Act) – NLEA (Nutrition Labeling and Education Act) – CGMP (Current Good Manufacture Practice: manufacturing, packaging, labeling, or holding operations for DS)	– 2002/46/EC – EC No 1924/2006	– Health Food Registration and Filing Regulation – Guidelines for the application of health food registration – Health Food Filing Work Guide – National food safety standard-Health foods GB 16740-2014 [23]	/
Compliance process		– The manufacturers and distributors are responsible – Notification/registration: DS containing a new ingredient	Notification/registration	– Registration: use the ingredients outside the raw material dictionary and excipient dictionary – Filing: use the ingredients listed in the raw material and excipient dictionary	/
Category	Foods	+	+	+	+
	Medicines	−	−	−	−
Manufacturer Registration		−	+ (Limited)	+	−
Presence of a positive list		−	+	+	NA
Good Manufacturing Practice (GMP)		+	+ (HACCP)	+	NA
Clinical trials of individual products		−	+ (New ingredients)	+ (New ingredients)	NA
Obligated to display the usage and dosage		+	+	+	NA
Serious adverse event reporting		+	+	−	NA
Labeling and packaging		+	+	+	NA
Shape description		+	+	Often	NA
Advertising requirements		+	+	+	NA
Health claims		−	−	−	NA

+/−: presence/absence of the factor; NA: not annotated.

**Table 4:** The prevalence of the most commonly used dietary supplements before and during the COVID-19 pandemic.

	Before the Pandemic (%)	During the Pandemic (%)	Type of Study	Country	References
*Vitamins*	27.7	58.0	Cross-sectional (online questionnaire)	Algeria	[49]
Vit. D	7.1	22.4			
Vit. C	19.8	53.1			
*Minerals*	18.4	50.0			
Zinc	4.6	44.9			
Magnesium	11.5	18.9			
Selenium	0.8	6.1			
*Others*	9.0	12.6			
Omega 3	4.2	9.7			
*Vitamins*			Cross-sectional (questionnaire-based)	Saudi Arabia	[51]
Vit. D	34.6	35.1			
Vit. C	48.8	68.4			
*Vitamins*			Cross-sectional (online survey)	Saudi Arabia	[50]
Vit. D	20.6	18.7			
Vit. C	12.5	14.9			
Multivitamin	24.6	44.9			
*Minerals*					
Zinc	1.3	4.6			
*Vitamins*			Cross-sectional (online survey)	Lebanon	[48]
Vit. D	35.5	41.0			
Vit. C	35.3	42.1			
Vit. E	15.2	17.5			
*Minerals*					
Zinc	18.8	29.3			
*Vitamins*			Cross-sectional	Turkey	[52]
Vit. D	10.7	5.5			
Vit. C	14.2	41.1			
*Minerals*					
Calcium	0.2	0.2			
Zinc	0.2	-			
